# Loss of heterozygosity of TRIM3 in malignant gliomas

**DOI:** 10.1186/1471-2407-9-71

**Published:** 2009-02-27

**Authors:** Jean-Louis Boulay, Urs Stiefel, Elisabeth Taylor, Béatrice Dolder, Adrian Merlo, Frank Hirth

**Affiliations:** 1 Department of Biomedicine, University Hospital, CH-4031 Basel, Switzerland; 2 Institute of Zoology and Biocenter, University of Basel, CH-4056 Basel, Switzerland; 3 MRC Centre for Neurodegeneration Research, King's College London, London, SE5 8AF, UK

## Abstract

**Background:**

Malignant gliomas are frequent primary brain tumors associated with poor prognosis and very limited response to conventional chemo- and radio-therapies. Besides sharing common growth features with other types of solid tumors, gliomas are highly invasive into adjacent brain tissue, which renders them particularly aggressive and their surgical resection inefficient. Therefore, insights into glioma formation are of fundamental interest in order to provide novel molecular targets for diagnostic purposes and potential anti-cancer drugs. Human *Tripartite motif protein 3 *(*TRIM3*) encodes a structural homolog of *Drosophila brain tumor *(*brat*) implicated in progenitor cell proliferation control and cancer stem cell suppression. *TRIM3 *is located within the loss of allelic heterozygosity (LOH) hotspot of chromosome segment 11p15.5, indicating a potential role in tumor suppression. ...

**Methods:**

Here we analyze 70 primary human gliomas of all types and grades and report somatic deletion mapping as well as single nucleotide polymorphism analysis together with quantitative real-time PCR of chromosome segment 11p15.5.

**Results:**

Our analysis identifies LOH in 17 cases (24%) of primary human glioma which defines a common 130 kb-wide interval within the *TRIM3 *locus as a minimal area of loss. We further detect altered genomic dosage of *TRIM3 *in two glioma cases with LOH at 11p15.5, indicating homozygous deletions of *TRIM3*.

**Conclusion:**

Loss of heterozygosity of chromosome segment 11p15.5 in malignant gliomas suggests *TRIM3 *as a candidate brain tumor suppressor gene.

## Background

Malignant gliomas are brain tumors that represent devastating and difficult-to-treat cancers with a mean patient survival of 10 months. Although surgical interventions allow resection and improve local tumor control, the further course of the disease remains dominated by re-appearance of unscheduled cell proliferation and insidious infiltration of normal brain tissue. Especially glioblastomas appear notoriously resistant to therapy, which has been attributed to DNA-repair proficiency and deregulated molecular pathways [1,2]. More recently combined chemoradiotherapy of concomitant and adjuvant temozolomide and radiotherapy has been introduced which can lead to significant prolongation of survival, in particular in patients with an epigenetically silenced DNA repair gene [3]. However, the outcome remains poor. This therapeutic resistance has recently been attributed to tumor stem-like cells due to their unrestrained self-renewal capacity and the ability to maintain tumorigenic potential at the single cell level, thereby evading both resection and radiotherapy [4-7]. There is growing evidence that some brain cancers arise either from normal stem cells or from progenitor cells in which self-renewal pathways have become aberrantly activated [7-10].

*Drosophila *Brain tumor (*brat*) has been identified as a regulator of progenitor cell proliferation control and cancer stem cell suppression [11-18]. Brat is expressed throughout *Drosophila *brain development and exerts an essential gate-keeper function in the binary switch between self-renewal and differentiation of neural progenitor cells. Neural progenitor cells mutated for Brat are unable to differentiate but rather continue to proliferate, resulting in *brat *mutant cells that display characteristic features of cancer-like stem cells. The resulting brain tumor tissue is characterized by pleiomorphic cells, continued proliferation and chromosome instability, as evidenced by a variety of karyotypic abnormalities [19].

Homologues of *brat *have been recorded in various species, with three human homologues, namely *Tripartite Motif Protein 2 *(*TRIM2*), *TRIM3*, and *TRIM32*, located on chromosome 4q31.3, 11p15.5, and 9q33.1, respectively [20-23]. 11p15 represents the telomeric end of chromosome 11 which shows loss of allelic heterozygosity (LOH) in various types of tumors, indicating the presence of one or more tumor suppressor genes [24-27]. Homologues of *TRIM3 *are primarily expressed in brain and may function at the interface of proliferation and differentiation during the maturation of brain tissue [28-30]. Here we report refined deletion mapping of chromosome 11p15.5 in malignant gliomas.

## Methods

### Biopsies and DNA extraction

Tumor samples used in this study were obtained from 70 patients who underwent glioma resection at the University Hospital of Basel between 1996 and 2005. The collection of tumor samples has been approved by the Ethics Committee of Basel-Land and Basel-Stadt (EKBB). Informed consent has been obtained together with the patient's permission to conduct open brain surgery, consenting to the use of biopsies for anonymous scientific research. This procedure follows the present recommendations of the Swiss Academy of Medical Sciences as proposed in 2008 and is in compliance with the Helsinki Declaration. Tumors were classified according to the revised WHO classification of tumors of the nervous system [31], comprising 10 oligodendrogliomas grade II, 13 astrocytomas grades I to III, and 47 glioblastoma multiforme [see Additional file 1]. DNA was extracted from fresh frozen primary gliomas and peripheral blood mononuclear cells (PBMCs) derived from the same patients, as previously described [32,33]. Only material containing less than 30% residual amounts of non-neoplastic cells was considered for further analysis.

### STS- and SNP-based LOH analysis

Sequence tagged site (STS)-based LOH was performed essentially as described [32]. Briefly, DNA from 70 glioma specimens and PBMCs of the same patients was analysed for loss of heterozygosity by amplification of microsatellite sequences [32]. Primers for these sequences were obtained from Microsynth (Balgach, Switzerland). Fluorescence based LOH mapping was employed with DNA from all gliomas. D11S4905 (located in *TRIM3 *intron 2) and D11S1250 primers were FAM-labeled, D1S1318 and D11S1758 primers were HEX-labeled, and D11S1331 and D11S1997 primers were TET-labeled. PCR product size fractionation and quantification were performed on ABI Prism 310 Genetic Analyzer (PE Applied Biosystems, Foster City, CA, USA). The ratio of peak heights of both alleles was calculated for each tumor and PBMC DNA sample. For informative cases, allelic loss was scored if the ratio between tumor and PBMC DNA was more than 1.5 (1/0) or less than 0.66 (0/2). Single nucleotide polymorphism (SNP)-based LOH was performed with the following markers: rs11605881 (*TRIM3 *promoter), rs11607224 (exon1), rs1060067 (exon4) rs16913748 (exon6), rs11605141 (intron6), rs13343175 (intron7), rs3830325 (intron9), rs2306897 (exon10) and rs2723636 (exon13). SNPs were visualized on an ABI Prism 310 Genetic Analyzer (PE Applied Biosystems, Foster City, CA, USA).

### Quantitative real-time PCR

Classification of the genetic status of *TRIM3 *was performed by quantitative real-time-PCR using the TaqMan ABI Prism^® ^7700 Sequence Detection System (PE Applied Biosystems, Foster City, CA, USA) as previously described [34]. Gene-specific primers for the reference-control *GAPDH*, which is located on chromosome 12p13.3, as well as primers for Intron1, Exon 3, Intron 6, Intron 11 and cDNA of *TRIM3 *were used as follows:

For *GAPDH*:

5'-AATGGGACTGAGGCTCCAC (sense),

5'-TTATGGGAAAGCCAGTCCCC (antisense).

For *TRIM3 *Intron 1:

5'-CCCCAAGGGTGCGTTTGTATT (sense),

5'-TGCTCTCACGGACATGGACA (antisense).

For *TRIM3 *Exon 3:

5'-GCAGTTCCTGGTATGCAGCAT (sense),

5'-TGCAGGCAAGGAAGAACCTT (antisense).

For *TRIM3 *Intron 6:

5'-GGGCCAAACAGAAGGTGTGT (sense),

5'-GGCATGTCAGGAGGCAGAAT (antisense).

For *TRIM3 *Intron 11:

5'-AGGCAGTAGGGCACATGGAT (sense),

5'-GAGAACCCCCACCCAGATCT (antisense).

For *TRIM3 *cDNA Exons 7/8:

5'-GGCGGCAAACGAAAGGA (sense),

5'-CCTTCCACGACTGCCAACA (antisense).

Gene-specific double-dye FAM-TAMRA labelled oligomeric probes were:

For *GAPDH*: FAM-ATCCAAGACTGGCTCCTCCCTGCTG-TAMRA.

For *TRIM3 *Intron 1: FAM-CCCACAGCCGCTCCGACCCA-TAMRA.

For *TRIM3 *Exon 3: FAM-TGCCTGGATCGGTACCAGTGCCC-TAMRA.

For *TRIM3 *Intron 6: FAM-CACCAGCTCCCCATTCCCCA-TAMRA.

For *TRIM3 *Intron 11: FAM-CAGCTACAGCCCAAATCTGCTTCATAGGCTT-TAMRA.

For *TRIM3 *cDNA: FAM-AACCCAATTGAGGATGAGCTCGTCTTCC-TAMRA.

PCR conditions, primers and probe design were assessed by the Primer Express^® ^program (PE Applied Biosystems, Foster City, CA, USA). Final primer concentration was 200 nM and fluorescent oligomeric probe concentration was 50 nM. Fifty ng genomic DNA derived from primary tumor tissue was taken as template. Genomic DNA amounts between normal and neoplastic tissues were standardized by subtracting the respective threshold cycles (C_t_s) obtained for the *GADPH *gene. In this approach the variable C_t _is defined as the fractional cycle number crossing a fixed threshold of fluorescence that has been generated by cleavage of the probe due to polymerase driven exonucleolytic activity. Differences in the C_t _values between two genes were referred to the ΔC_t _value. After normalization of the tumor C_t _values with the PBMC C_t _values, the ΔC_t_values and the relative copy number of Introns1, 6 and 11, as well as Exon 3 of *TRIM3 *were calculated as follows: ΔC_t _(X) = C_t _(reference) - C_t _(X). Thus, ΔC_t _values of 0 ± 0.3 [-0.3;+0.3] indicated retention of both alleles (diploidy = 2n) and ΔC_t _values of -1 ± 0.3 [-0.7;-1.3] indicated loss of one allele (haploidy = n). Due to the frequent contamination of tumor biopsy DNA by that of invaded normal tissue, ΔC_t _values of < -1.30 were accounted as indicative for homozygous deletions. All analyses of tumor samples were performed in triplicate in parallel with PBMC DNA, and data indicating homozygous deletions resulted from two independent experiments.

## Results

### LOH in human gliomas reveals a 130 kb minimally lost area uncovering *TRIM3*

**Figure 1:**
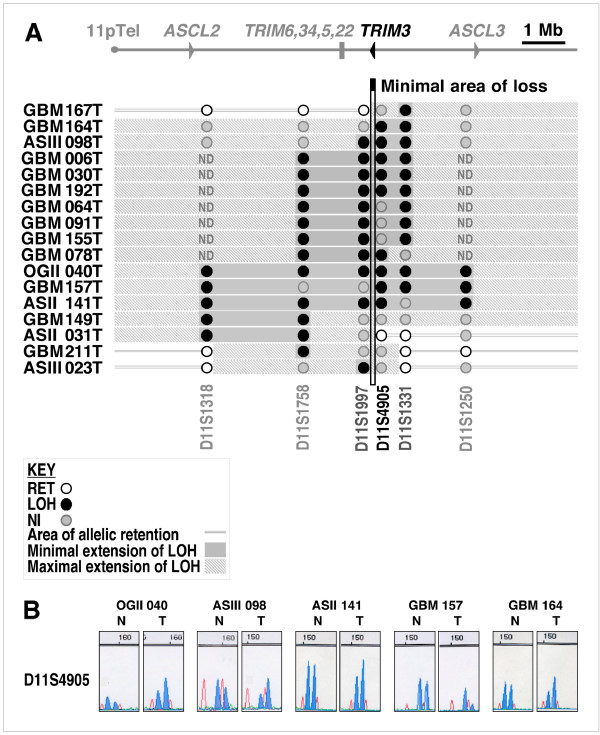
**Deletion mapping of chromosomal region 11p15.5 in human primary gliomas defines a 130 kb-wide area containing *TRIM3***. **A**. The 11p15.5 chromosomal region highlighting *TRIM3*, the more distantly related *TRIM5*, *TRIM6*, *TRIM22*, and *TRIM34 *genes, as well as *ASCL2 *and *ASCL3*; arrows indicate transcription orientations. Middle panel: somatic deletion of the 11p15.5 region in glioma. Tumor histology, grades and numbers are indicated on the left. Allelic retention, allelic loss and non-informative data are represented by open, closed and grey circles, respectively. Minimal and maximal extensions of allelic loss are shown with filled and hatched grey areas, respectively. The 11p15 microsatellite markers/sequence tag sites (STS) used are indicated (D11S1318, D11S1758, D11S1997, D11S4905, D11S1331, D11S1250). Alignment of LOH data delimits a minimal area of 130 kb showing LOH common to all 10 tumors between markers D11S1997 and D11S4905 which identifies the *TRIM3 *locus. GBMs displaying complete loss of the central region were not tested for D11S1318 and D11S1250 (ND). **B**. Informative LOH data with marker D11S4905 comparing normal (N) with tumor (T) DNA for brain tumors OGII 040, ASIII 098, ASII 141, GBM 157, GBM 164. Abbreviations: GBM, Glioblastoma multiforme; AS, astrocytoma; OG, oligodendroglioma; RET, retention; LOH, loss of heterozygosity; ND, not determined; NI, non-informative.

Initial studies had identified an approximately 21 centiMorgan region on 11p15.5-pter that showed frequent loss of heterozygosity in malignant gliomas [35]. This region has been refined to 7 megabases (Mb) [24], spanning the region 11p15.4–5 between microsatellite markers (also called STS) D11S922 and D11S1250 (Fig. 1). Several genes are contained within this interval that might be involved in brain development and/or tumor suppression, including *TRIM3 *and a cluster of genes encoding the more distantly related *TRIM5*, *TRIM6*, *TRIM22*, and *TRIM34 *genes. This area also contains *ASCL2 *and *ASCL3*. Both genes are homologues of the *Drosophila *genes of the *achaete-scute *(ASC) complex that promote cells to develop a neural fate [36], which therefore may represent additional candidate brain tumor suppressors encoded by this region (Fig. 1). We performed loss of heterozygosity analysis on human brain tumor samples representing 70 primary gliomas of varying histology and grades including 13 astrocytomas (AS) WHO grades I to III, 10 oligodendrogliomas (OG) WHO grade II, and 47 glioblastomas (GBM). We focused our LOH analysis on the region spanning *TRIM3 *by using 6 microsatellite markers in addition to the ones that had been used earlier to determine the minimal area of loss [24].

Among the 70 primary tumor samples tested, 17 out of 70 (24%) showed LOH of at least one marker in this region. Heterozygous deletions included 12 GBM out of 47 analysed (25%) consistent with the 11p15.5 deletion frequency previously described in GBM [24]. Further, one OG grade II out of 10 (10%) showed 11p15.5 allelic loss. No allelic loss was detected in AS grade I (0/3), while heterozygous deletions were observed in 2 AS grade II out of 7 (28%) as well as in 2 AS grade III out of 3 (67%), suggesting a graded escalation of 11p15.5 deletion frequency with tumor grading in AS (see Table 1 and Fig. 1). Some of these heterozygous deletions, including OGII 040, ASII 141, and GBM 157, covered the complete region 11p15 (D11S1318, D11S1758, D11S1997, D11S4905, D11S1331, D11S1250). At the telomeric side, GBM tumor 167 showed the longest retention proximally extending to STS marker D11S1997, whereas ASII tumor 031 had the most distal extension of retention to STS marker D11S4905. Therefore, alignment of our LOH data with the physical map of the 11p telomeric region delimited a minimal area of loss common to all of these tumors between markers D11S1997 and D11S4905. This interval reduced the minimal area of loss from 7 Mb to only 130 kilobases (kb) covering the *TRIM3 *locus (Fig. 1), and also pointed to potential breakpoint mutations within the *TRIM3 *gene between Exons 3–13.

**Table 1:** 11p15.5 LOH frequencies among glioma subsets

Histology	Number	LOH	no LOH	% LOH
OG II	10	1	9	10
AS I	3	0	3	0
AS II	7	2	5	28
AS III	3	2	1	67
GBM	47	12	35	25
∑	70	17	53	24

Abbreviations: OG, oligodendroglioma; AS, astrocytoma; GBM, Glioblastoma multiforme; I-III, WHO grade; LOH, loss of heterozygosity.

### Genomic dosage alterations of *TRIM3 *in malignant gliomas

**Figure 2:**
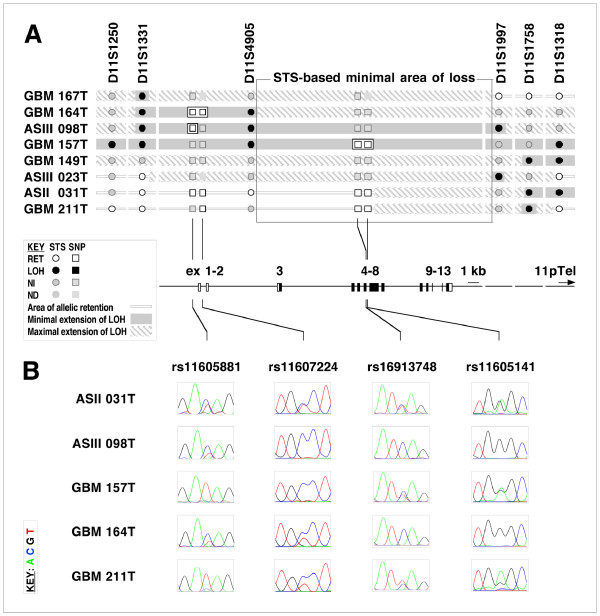
**SNP-based somatic deletion mapping of chromosomal region 11p15.5 identifies potential breakpoint mutations within the *TRIM3 *gene**. **A**. Superimposition of single nucleotide polymorphism (SNP) and sequence tag site (STS)-based LOH data. (Note that compared to Fig. 1, the map has been inverted to comply with the 5'-3' transcription orientation of the *TRIM3 *gene). STS markers (D11S series) are indicated on the top; tumor histology, grades and numbers are indicated on the left. Allelic retention, allelic loss and non-informative data are represented by open, closed and grey circles, respectively. Minimal and maximal extension of STS-based areas of allelic loss described in Fig. 1, are shown with filled and hatched grey areas, respectively. Areas of local allelic retention defined by SNP analysis within segments of allelic loss defined by STS analysis are framed by open rectangles. SNP and STS data, as well as SNP markers (rs series) used are related to a structural map of the *TRIM3 *gene with its coding regions shown in black (bottom). Thus, SNP-based allelic retention of short sections within the areas of LOH in primary gliomas ASIII 098, GBM 157, and GBM 164 suggest potential homozygous deletions within the *TRIM3 *gene. **B**. SNP bi-allelism in primary tumors with 11p15.5 allelic loss. Sequence electrophoretograms of genomic DNA extracted from primary tumor samples amplified at indicated SNP markers of the *TRIM3 *gene area; peak color codes: green (A), blue (C), black (G), red (T). Abbreviations: GBM, Glioblastoma multiforme; AS, astrocytoma; OG, oligodendroglioma; RET, retention; LOH, loss of heterozygosity; NI, non-informative.

In order to refine somatic deletion mapping and to delimit the minimal area of loss in more detail, we used single nucleotide polymorphic (SNP) markers located within the *TRIM3 *genomic area to further investigate a selection of tumor samples. For further analysis, we selected those tumors that were indicative for loss or at least partial loss of the analysed region at 11p15, namely GBMs 149, 157, 164, 167, and 211, as well as ASII 031, ASIII 023, and ASIII 098 (Fig. 2A).

Among the ten SNPs initially selected, six turned out to be non-informative in all analyzed tumors whereas four SNPs, namely rs11605881, rs11607224, rs16913748, rs11605141, displayed loss of heterozygosity or allelic retention in those tumors selected for further analysis (Fig. 2). Thus, we observed allelic retention of both parental alleles of SNPs rs11605881, rs16913748, and rs11605141 in primary tumors ASII 031 and GBM 211, respectively. These data displaced the centromeric rim of the minimally lost area of *TRIM3 *from STS D11S4905 to SNP rs11605141 but still targeted the *TRIM3 *gene (Fig. 2). In addition, in those cases where we observed STS-based loss of heterozygosity extending on both sides of the *TRIM3 *gene (ASIII 098, GBM 157 and GBM 164), the detected area of LOH was locally interrupted by short sections with allelic retention at SNPs rs11605881 and rs11607224. Indeed, tumors ASIII 098 and GBM 164 showed heterozygosity at SNP markers rs11605881 and rs11607224 located in the *TRIM3 *promoter and in exon1, respectively, whereas analysis of markers rs16913748 and rs11605141 of *TRIM3 *intron 6 revealed heterozygosity in GBM 157 (Fig. 2).

Allelic retention within a chromosomal interval displaying LOH has been interpreted as a potential site of homozygous deletion, where retention seems to result from the amplification of wildtype DNA deriving from non-neoplastic cells present in the tumor biopsy [37]. Thus, SNP-based allelic retention of short sections within the areas of LOH in primary gliomas ASIII 098, GBM 157, and GBM 164 indicated potential homozygous deletions within the *TRIM3 *gene. In order to investigate this possibility, we targeted four equidistant regions of the *TRIM3 *gene, including the two areas of possible homozygous loss in the three primary tumor samples ASIII 098, GBM 157 and GBM 164 by quantitative real-time PCR (Q-PCR). Analysis of the genetic status of *TRIM3 *in ASIII 098, GBM 157, and GBM 164 was assayed on DNA extracted from both the primary gliomas and peripheral blood mononuclear cells (PBMCs) derived from the same patients by Q-PCR of the reference-control gene GAPDH, as well as for Intron1, Exon 3, Intron 6, and Intron 11 of *TRIM3*, respectively (see Methods).

**Figure 3:**
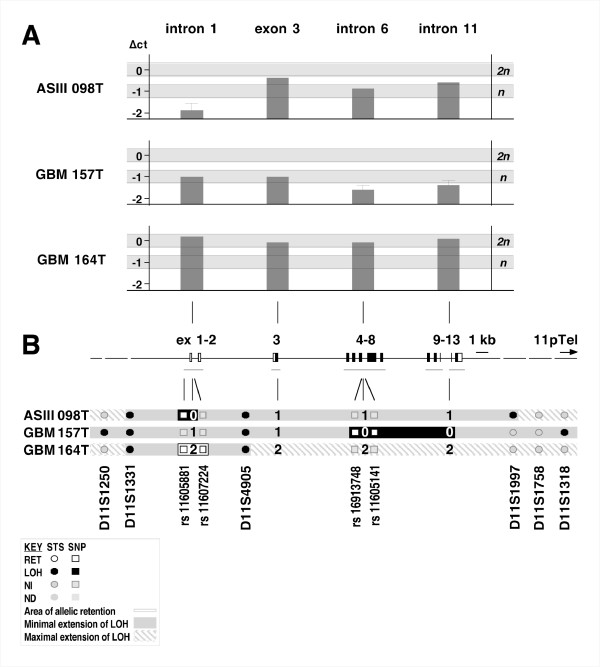
**Altered genomic dosage of *TRIM3 *suggests homozygous deletions in human primary gliomas**. **A**. Genomic dosage of the TRIM3 gene area of ASIII 098, GBM 157, and GBM 164 as determined by quantitative real-time PCR. Light grey background indicates ΔC_t _cut-offs for diploidy (2n = 0 ± 0.3) and haploidy (n = -1 ± 0.3) for each primary tumor sample; grey bars depict ΔC_t _values of amplified tumor DNA related to a structural map of the *TRIM3 *gene with coding regions shown in black **B**. Mapping of indicated homozygous deletions within the TRIM3 gene. STS-based and SNP-based compilation of Q-PCR data is shown in relation to structural map of the *TRIM3 *gene with coding regions shown in black. Tumor histology, grades and numbers are indicated on the left. STS-based (D11S series) allelic loss and non-informative data are represented by closed and grey circles, respectively. Areas of local allelic retention defined by SNP analysis (rs series) within segments of allelic loss defined by STS analysis are framed by open rectangles. Copy number deduced from Q-PCR data are indicated; minimal and maximal extension of allelic loss' are shown with filled and hatched grey areas, respectively; areas of homozygous loss are shown in black. Q-PCR data indicate homozygous deletions in primary tumors ASIII 098 and GBM 157. Abbreviations: GBM, Glioblastoma multiforme; AS, astrocytoma; OG, oligodendroglioma; RET, retention; LOH, loss of heterozygosity; NI, non-informative; Q-PCR, quantitative real-time polymerase chain reaction.

In ASIII 098, tumor genomic dosage in *TRIM3 *intron 1, at the site between SNP markers rs11605881 and rs11607224 indicated DNA levels below haploidy of ΔC_t _= -1.98 ± 0.40 (for calculation details, see Methods). These results signified a homozygous deletion encompassing *TRIM3 intron 1 *as already indicated by data of SNP rs11605881 analysis (Fig. 3). Similarly, Q-PCR analysis of GBM 157 indicated DNA levels below haploidy from *TRIM3 intron 6 *(ΔC_t _= -1.59 ± 0.20) to *intron 11 *(ΔC_t _= -1.43 ± 0.20), signifying homozygous deletion as already indicated by SNPs markers rs1693748 and rs1605141 (Fig. 3). In contrast, genomic dosage of primary tumor GBM 164 indicated continuous diploidy along the *TRIM3 *gene as exemplified by ΔC_t _values between -0.30 and +0.30 of four Q-PCR markers covering intron 1, exon 3, intron 6 and intron 11 (Fig. 3). Thus, among the 10 primary human glioma identified with allelic loss at 11p15.5, Q-PCR analysis of ASIII 098 and GBM 157 (20%) indicated homozygous deletions within the *TRIM3 *gene.

## Discussion

In cancer research, conventional strategies of somatic deletion mapping rely on the detection of frequent sites of larger DNA alterations. Accordingly, allelic loss of heterozygosity analysis of tumor DNA facilitates the identification and localization of a minimally lost area correlating with candidate tumor suppressor gene loci that might be involved in the tumorigenic process [38]. In contrast to these conventional *top-down *strategies, we carried out somatic deletion mapping on human glioma DNA focusing on the *TRIM3 *locus. We opted for this unorthodox *bottom-up *strategy as a result of significant structural homologies between TRIM3 and the *Drosophila *brain tumor suppressor Brat [19], as well as owing to the fact that *TRIM3 *is located on chromosome segment 11p15.5.

Previous analyses demonstrated that chromosome segment 11p15 contains a region of frequent loss of allelic heterozygosity in various adult tumors including those of the brain, lung, breast, ovary, esophagus, stomach, as well as others [27,35,39-44]. The frequency of LOH in this region and its apparent correlation with metastatic tumor spread suggests that this chromosome segment may represent a hotspot containing one or more tumor suppressor gene(s).

In the case of brain tumors, a previous LOH study delimited the minimal area of loss to a final 7 Mb-wide genomic interval spanning several genes with a potential role in both developing and neoplastic brain tissue [24]. Among those genes are *ASCL2 *and *ASCL3*, mammalian homologues of *Drosophila achaete-scute *complex [36], as well as *TRIM3*, a homologue of *Drosophila brain tumor *involved in progenitor cell proliferation control and cancer stem cell suppression [19], and a cluster of more distantly related TRIM genes (see Figure 1).

Compared to the earlier study by Schiebe *et al*. [24], we analyzed an equivalent number of GBMs (n = 47 vs. n = 50), as well as brain tumors that are distinct in origin, i.e. OG (n = 10) and AS (n = 13). By focusing on GBM only (see Table 1), our data revealed similar deletion frequencies (25% vs. 28%) to those previously obtained in GBM [24]. Interestingly, our deletion analysis revealed increased frequencies in AS of higher grade, which might indicate a possible association of 11p15.5 allelic loss with tumor progression. However, this conclusion needs to be strengthened by the analysis of a larger number of AS.

Furthermore, as compared to the 7 Mb region previously described [24], our somatic deletion mapping analysis delimited a 130 kb-wide minimal area of loss. Thus, our results rule out *ASCL2 *and *ASCL3 *and the more distantly related *TRIM *genes as potential glioma tumor suppressors within the genomic region analyzed. Significantly, the 130 kb-wide minimal area of loss not only identified the *TRIM3 *locus, but also indicated potential breakpoint mutations within the *TRIM3 *gene.

We further substantiated our LOH data by single nucleotide polymorphism analysis together with quantitative real-time PCR. Previous studies showed that Q-PCR can identify micro-deletions providing a reliable approach for a direct and specific determination of the ploidy status within defined genetic loci. This approach led to the identification of homozygous deletions of the *p14ARF/p16INK4a *tumor suppressor locus which is frequently affected in human gliomas [34]. Significantly, we detected genomic dosage alterations of *TRIM3 *in two glioma cases with LOH at 11p15.5, indicating homozygous deletions of *TRIM3*. Our LOH and Q-PCR data therefore suggest that TRIM3 may act as a tumor suppressor in the human brain. However, in vitro and mammalian in vivo loss- as well- as gain-of function analyses are required to determine the function of TRIM3 in detail. It will be interesting to see whether TRIM3, similar to its *Drosophila *homologue Brain tumor, is involved in the regulation of progenitor cell proliferation control and brain tumor suppression.

## Conclusion

Our analysis identifies loss of allelic heterozygosity at 11p15.5 in 17 cases of primary human glioma and defines a common 130 kb-wide interval as a minimal area of loss that covers the *TRIM3 *locus. In two glioma cases with LOH, altered genomic dosage of *TRIM3 *indicates homozygous deletions. Together, these data suggest TRIM3 as a 11p15.5 candidate brain tumor suppressor gene. Further investigation will be needed to elucidate the biological function of TRIM3 and its precise role in brain tumor suppression.

## Abbreviations

GBM: Glioblastoma multiforme; AS: astrocytoma; OG: oligodendroglioma; RET: retention; LOH: loss of heterozygosity; ND: not determined; NI: non-informative; Q-PCR: quantitative real-time polymerase chain reaction.

## Competing interests

The authors declare that they have no competing interests.

## Authors' contributions

FH conceived, and FH, JLB and AM designed the study; US, ET, and BD acquired the data; FH, JLB and AM analyzed and interpreted the data; FH and JLB wrote the manuscript. All authors read and approved the final manuscript.

## Pre-publication history

The pre-publication history for this paper can be accessed here:

http://www.biomedcentral.com/1471-2407/9/71/prepub

## Supplementary Material

Additional file 1 LOH data for all gliomas investigated. The data provided represent all 70 glioma cases investigated. Click here for file
