# Supplementary material for: Loss of heterozygosity of TRIM3 in malignant gliomas
Source: BMC Cancer. 2009 Feb 27;9:71. doi: 10.1186/1471-2407-9-71 (PMC2653542; doi:10.1186/1471-2407-9-71)
Supplement: Additional file 1 — LOH data for all gliomas investigated. The data provided represent all 70 glioma cases investigated. [file 1471-2407-9-71-S1.pdf]

Boulay et al: Loss of heterozygosity of TRIM3 in malignant glioma

Additional file 1 - LOH data for all gliomas investigated

Gliomas are anonymously numbered according to patient data. The 11p15 microsatellite markers / sequence tag sites used are indicated (D11S1318, D11S1758, D11S1997, D11S4905, D11S1331, D11S1250). Abbreviations: GBM, Glioblastoma multiforme; AS, astrocytoma; OG, oligodendroglioma; ND, not determined; RET, retention; LOH, loss of heterozygosity; NI, non-informative; I-III, WHO grade.

tumor#	type/grade	D11S1318	D11S1758	D11S1997	D11S4905	D11S1331	D11S1250
4	GBM	ND	RET	RET	RET	NI	ND
5	GBM	RET	RET	RET	RET	RET	NI
6	GBM	ND	LOH	LOH	LOH	LOH	ND
11	GBM	ND	NI	RET	RET	RET	ND
29	GBM	ND	RET	RET	RET	RET	ND
30	GBM	ND	LOH	LOH	LOH	LOH	ND
38	GBM	RET	NI	RET	NI	RET	RET
45	GBM	ND	RET	RET	NI	RET	ND
47	GBM	ND	NI	RET	RET	RET	ND
56	GBM	ND	RET	RET	RET	RET	ND
59	GBM	RET	RET	NI	RET	RET	RET
60	GBM	ND	RET	NI	RET	RET	ND
64	GBM	ND	LOH	NI	LOH	LOH	ND
69	GBM	RET	RET	NI	RET	RET	RET
70	GBM	ND	RET	RET	RET	RET	ND
72	GBM	RET	RET	RET	RET	NI	NI
78	GBM	ND	LOH	LOH	LOH	NI	ND
91	GBM	ND	LOH	LOH	NI	LOH	ND
93	GBM	NI	NI	RET	RET	RET	RET
116	GBM	RET	NI	RET	NI	RET	RET
117	GBM	RET	RET	RET	RET	NI	RET
128	GBM	NI	RET	RET	RET	RET	RET
132	GBM	NI	RET	RET	NI	RET	NI
138	GBM	RET	RET	RET	RET	NI	NI
143	GBM	NI	NI	RET	RET	RET	NI
145	GBM	RET	RET	RET	RET	RET	NI
146	GBM	RET	NI	RET	RET	RET	RET

**tumor#**	**type/grade**	**D11S1318**	**D11S1758**	**D11S1997**	**D11S4905**	**D11S1331**	**D11S1250**
149	GBM	LOH	LOH	NI	NI	NI	NI
153	GBM	RET	RET	RET	NI	RET	RET
155	GBM	ND	LOH	NI	LOH	LOH	ND
154	GBM	ND	ND	RET	NI	RET	RET
157	GBM	LOH	LOH	LOH	LOH	LOH	LOH
161	GBM	RET	NI	RET	NI	RET	NI
163	GBM	NI	NI	RET	RET	RET	RET
164	GBM	NI	NI	NI	LOH	NI	NI
167	GBM	RET	RET	RET	NI	LOH	NI
170	GBM	ND	NI	NI	RET	RET	ND
171	GBM	RET	NI	RET	NI	RET	RET
101	ASI	RET	RET	RET	NI	RET	RET
51	ASII	NI	RET	RET	RET	NI	NI
61	ASII	LOH	LOH	NI	RET	RET	NI
85	ASII	RET	RET	NI	RET	RET	RET
120	ASII	RET	RET	NI	RET	RET	NI
141	ASII	LOH	LOH	LOH	LOH	NI	LOH
152	ASII	NI	RET	RET	RET	RET	RET
159	ASII	RET	RET	RET	NI	NI	RET
23	ASIII	RET	NI	LOH	NI	RET	NI
67	ASIII	RET	NI	RET	RET	RET	NI
98	ASIII	NI	NI	LOH	LOH	LOH	NI
22	OGII	NI	RET	RET	RET	RET	RET
40	OGII	LOH	LOH	LOH	LOH	LOH	LOH
52	OGII	NI	RET	RET	NI	NI	RET
77	OGII	RET	RET	RET	RET	NI	RET
79	OGII	NI	RET	RET	RET	NI	RET
87	OGII	RET	NI	NI	RET	NI	NI
92	OGII	RET	RET	RET	RET	NI	NI
112	OGII	RET	RET	RET	RET	RET	NI
115	OGII	RET	RET	RET	RET	NI	NI
169	OGII	RET	RET	RET	NI	RET	RET
